# Cloning and characterization of the pepper *CaPAO* gene for defense responses to salt-induced leaf senescence

**DOI:** 10.1186/s12896-015-0213-1

**Published:** 2015-10-24

**Authors:** Huai-Juan Xiao, Ke-Ke Liu, Da-Wei Li, Mohamed Hamed Arisha, Wei-Guo Chai, Zhen-Hui Gong

**Affiliations:** College of Horticulture, Northwest A&F University, Yangling, Shaanxi P. R. China; College of Horticulture, Henan Agricultural University, Zhengzhou, Henan P. R. China; Faculty of Agriculture, Zagazig University, Zagazig, Sharkia P. R. Egypt; Institute of Vegetables, Hangzhou Academy of Agricultural Sciences, Hangzhou, Zhejiang P. R. China

**Keywords:** *Capsicum annuum* L, *CaPAO* gene, Expression analysis, Functional analysis, Tobacco

## Abstract

**Background:**

Pheophorbide a oxygenase (PAO) is an important enzyme in the chlorophyll catabolism pathway and is involved in leaf senescence. It opens the porphyrin macrocycle of pheophorbide *a* and finally forms the primary fluorescent chlorophyll catabolite. Previous studies have demonstrated the function of PAO during cell death. However, the characterizaton of PAO during leaf senescence induced by environmental factors is not well understood.

**Methods:**

Homology-based cloning and RACE techniques were used to obtain the full-length cDNA of the *CaPAO* gene. *CaPAO* expression was determined by quantitative real-time PCR. Function of *CaPAO* gene were studied using virus-induced gene silencing and transgenic techniques with tobacco plants (*Nicotiana tabacum*).

**Results:**

A novel PAO gene *CaPAO* was isolated from pepper (*Capsicum annuum* L.). The full-length *CaPAO* cDNA is comprised of 1838 bp, containing an open reading frame of 1614 bp, and encodes a 537 amino acid protein. This deduced protein belongs to the Rieske-type iron-sulfur superfamily, containing a conserved Rieske cluster. *CaPAO* expression, as determined by quantitative real-time PCR, was higher in leaves than roots, stems and flowers. It was upregulated by abscisic acid, methyl jasmonate and salicylic acid. Moreover, *CaPAO* was significantly induced by high salinity and osmotic stress treatments and also was regulated by *Phytophthora capsici*. The virus-induced gene silencing technique was used to silence the *CaPAO* gene in pepper plants. After 3 days of high salt treatment, the chlorophyll breakdown of *CaPAO*-silenced pepper plants was retarded. RD29A promoter-inducible expression vector was constructed and transferred into tobacco plant. After 7 days of salt treatment, the leaves of transgenic plants were severely turned into yellow, the lower leaves showed necrotic symptom and chlorophyll content was significantly lower than that in the control plants.

**Conclusions:**

The expression of *CaPAO* gene was induced in natural senescence and various stresses. The *CaPAO* gene may be related to defense responses to various stresses and play an important role in salt-induced leaf senescence.

## Background

Leaf senescence is the final stage of leaf development, ultimately leading to the death of the entire leaf. Although governed by the developmental age, it also can be stimulated by diverse environmental factors, including plant hormones, drought, salinity, extreme temperature, darkness, wounding and pathogenic infection [1, 2]. Premature leaf senescence can eventually affect the yield of plants under adverse environmental conditions. Therefore, studying leaf senescence will not only strengthen our comprehension of a basic biological process, it also may provide methods to delay plant aging in order to improve agricultural traits of vegetable crops. Pepper (*Capsicum annuum* L.) is an important vegetable crop that is extensive cultivated worldwide. In recent years, premature senescence of pepper plants caused by various environmental stresses has become a universal phenomenon and considered an important field of research.

Loss of green color, induced by degradation of chlorophyll, is the most visible symptom of leaf senescence. Pheophorbide *a* oxygenase (PAO) has been regarded as a crucial enzyme in chlorophyll degradation [3, 4]. It oxygenolytically cleaves the porphyrin macrocycle of pheophorbide (pheide) *a* and finally forms the primary fluorescent chlorophyll catabolite (FCC). The *PAO* gene was initially obtained from maize (designated *ZmLls1*) and then discovered in other higher plants, such as rice, wheat, tomato, soybean and canola [5–9]. Previous studies showed that the expression of *PAO* is induced by natural senescence and environmental stresses in plants [10–12]. In *Arabidopsis*, AtPAO is encoded by the *accelerated cell death 1* (*ACD1*) gene and is homologous to *lethal leaf spot 1* (*LLS1*) of maize. It belongs to a small family of Rieske-type iron-sulfur oxygenases [13]. The absence of *ACD1* has been shown to result in the accumulation of PAO and light-independent cell death when senescence is induced in permanent darkness [14, 15].

Tobacco (*Nicotiana tabacum*) is one of the most important model plants for transgenic research. In previous studies, many new genes from other plants have been transferred into tobacco for further research on their functions [16, 17]. The *RD29A* promoter from *Arabidopsis thaliana* is a stress-inducible promoter. This promoter contains two dehydration- responsive elements (DREs), which are involved in response to salt, dehydration and low temperature [18]. Using the *RD29A* promoter instead of the constitutive 35S CaMV promoter for certain genes overexpression minimizes the negative effects on plant growth, which have been widely used in genetic transformation [16].

In recent years, the research on PAO focused on the functional analysis for inhibiting cell death [11, 13, 14]. However, little is known about *CaPAO* regulatory role after induction expression of inducible-promoter in pepper. This study was conducted to clone this gene from the pepper plant and analyze its molecular characteristics. Patterns of *CaPAO* gene expression in specific tissues and in response to various stresses were analyzed by quantitative real-time PCR. Furthermore, virus-induced gene silencing (VIGS) and transgenic technologies were used to study *CaPAO* gene function. The results suggest that *CaPAO* may play an important role during leaf senescence and chlorophyll degradation.

## Results

### Cloning and Sequence Analysis of *CaPAO*

**Fig. 1:**
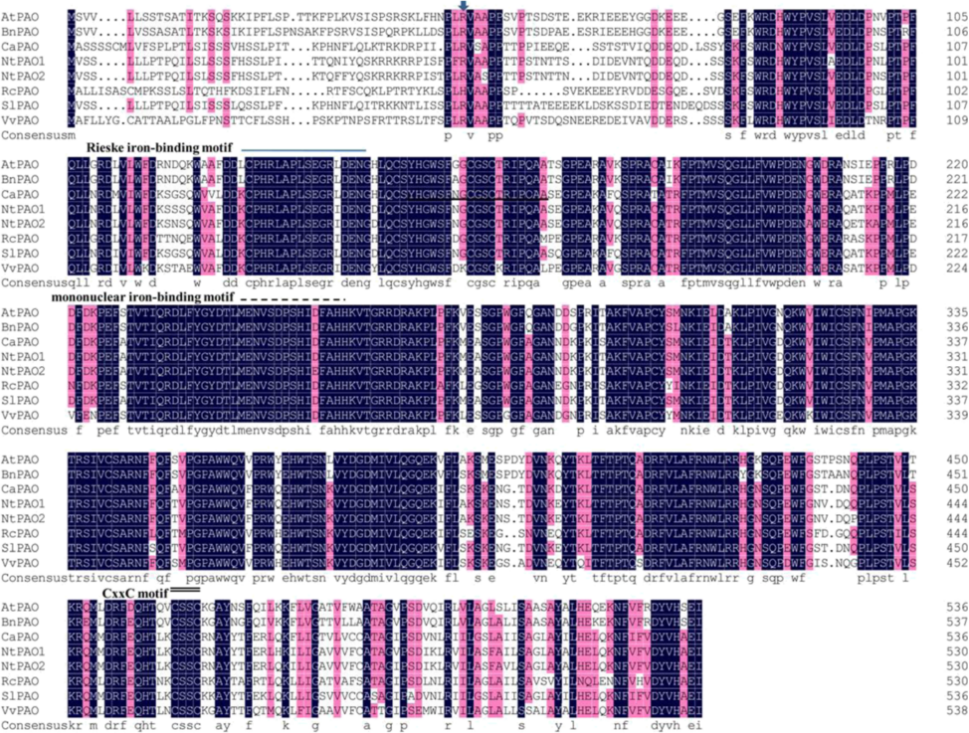
Multiple sequence alignment of the CaPAO protein and PAO proteins from other plants using DNAMAN software. The arrow indicates the cleavage site of the chloroplast peptide, and the single line indicates the Rieske iron-binding motif. The dashed line indicates the mononuclear iron-binding motif, and a conserved CxxC motif is indicated by a double line. Besides *Ca*PAO, other amino acid sequences included in this alignment were *S. lycopersicum* lethal leaf spot 1-like protein (NP_001234535), *N. tabacum* PAO1 (ABY19384.1), *N. tabacum* PAO2 (ABY19385.1), *V. vinifera* PAO (ACO56118.1), *R. communis* PAO (XP_002523735.1), *B. napus* PAO (ABD60317.1), and *A. thaliana* PAO (AEE77964.1). Shaded regions show identical amino acid residues among all species

The full-length cDNA designated *CaPAO* was obtained using *in silico* cloning and RACE techniques. The transcript consists of 1838 nucleotides, including a 5′-untranslated region (UTR) of 32 bp, an ORF of 1614 bp and a 3′-UTR of 192 bp (GenBank accession number KC176709) (Fig. 1). *CaPAO* was predicted to encode a 537 amino acid protein with a theoretical molecular weight (MW) of 60.8 kDa and calculated isoelectric point (p*I*) of 6.89. Structural analysis revealed that *CaPAO* belongs to a Rieske-type iron-sulfur superfamily, containing a conserved Rieske cluster, a mononuclear iron-binding site and a redox-active CxxC motif in the C-terminal end, which are necessary for oxygen activation (Fig. 1). Subcellular analysis localized *CaPAO* in the chloroplasts. Further sequence analysis indicated that the deduced *CaPAO* protein contains a chloroplast transit peptide of 50 amino acid residues with a cleavage site located between R_50_ and V_51_, but it does not contain a signal peptide region or transmembrane helix.

The deduced *CaPAO* amino acid sequence showed high homology to other plant PAO sequences via multiple alignments using DNAMAN software (Fig. 1). The percent identities of CaPAO relative to *Solanum lycopersicum* lethal leaf spot 1-like protein (NP_001234535), *N. tabacum* PAO1 (ABY19384.1) and PAO2 (ABY19385.1), *Vitis vinifera* PAO (ACO56118.1), *Ricinus communis* PAO (XP_002523735.1), *Brassica napus* PAO (ABD60317.1) and *A. thaliana* PAO (AEE77964.1), were 90, 85, 88, 76, 75, 71 and 71 %, respectively.

**Fig. 2:**
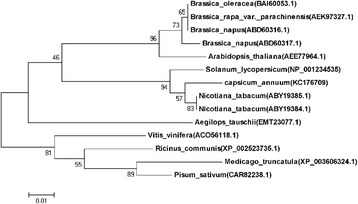
Phylogenetic analysis of CaPAO protein and PAO proteins of other plant species. The phylogenetic tree was constructed by the neighbor-joining method using MEGA5.05. Branches were labeled with the names and GenBank accession numbers of the different plant species

A phylogenetic tree, constructed using MEGA5.05 software, was used to investigate the evolutionary relationship of the CaPAO amino acid sequence with PAO proteins of other plants. Two groups were formed using the 14 PAO protein sequences from *C. annuum* L., *N. tabacum*, *S. lycopersicum*, *R. communis*, *V. vinifera*, *B. napus*, *A. thaliana*, *Medicago truncatula*, *Aegilops tauschi*, *Pisum sativum* and *Brassica rapa* var. parachinensis (Fig. 2)*.* CaPAO clustered in the first group, which included SlLLS1, NtPAO, BoPAO, BrPAO and AtPAO. CaPAO was more closely related to SlLLS1, NtPAO than PAO proteins of other plants. All of the above-mentioned bioinformatic analyses demonstrated that CaPAO should function as a PAO.

### Tissue-Specific Expression of *CaPAO*

**Fig. 3:**
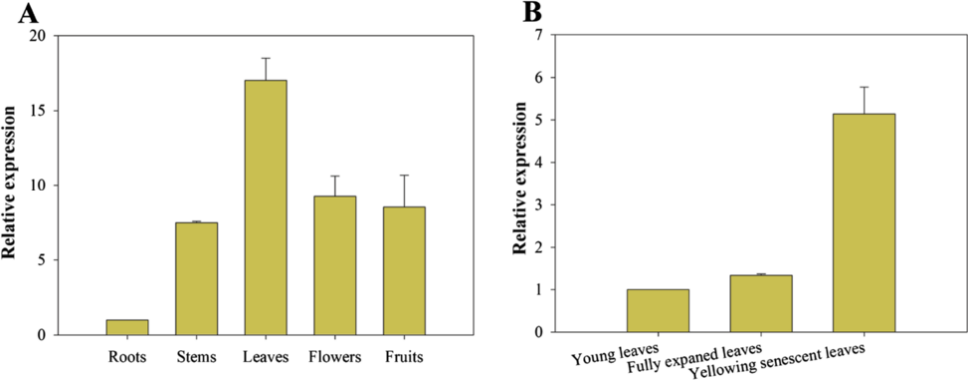
Tissue-specific expression of *CaPAO* in pepper. **a** Tissue-specific expression of *CaPAO* in pepper roots, leaves, stems and flowers. Relative expression levels of the *CaPAO* transcript were determined in different tissues in comparison to that in roots; **b** The expression profiles of *CaPAO* during pepper leaf developmental stage. RNA was extracted from young leaves, fully expanded leaves and senescent leaves, respectively. Error bars represent SD for three independent replicates

In order to investigate the expression levels of the *CaPAO* gene in different tissues, total RNA was extracted from the roots, stems, leaves and flowers, and quantitative real-time PCR (qRT-PCR) was performed (Fig. 3a). *CaPAO* transcripts were detected in all of these tissues and found to be higher in leaves than in other tissues. For different leaf developmental stages, a low level of *CaPAO* transcript was detected in young and fully mature leaves, but *CaPAO* expression was increased in senescent leaves (Fig. 3b).

### Induction of signaling molecules, abiotic and biotic stresses

In order to study its function in pepper plants, the expression pattern of *CaPAO* was first analyzed. B12 cultivar seedlings at the six-leaf stage were treated with various stresses, including phytohormones, salt, osmosis and *Phytophthora capsici* infection, and analyzed by qRT-PCR.

**Fig. 4:**
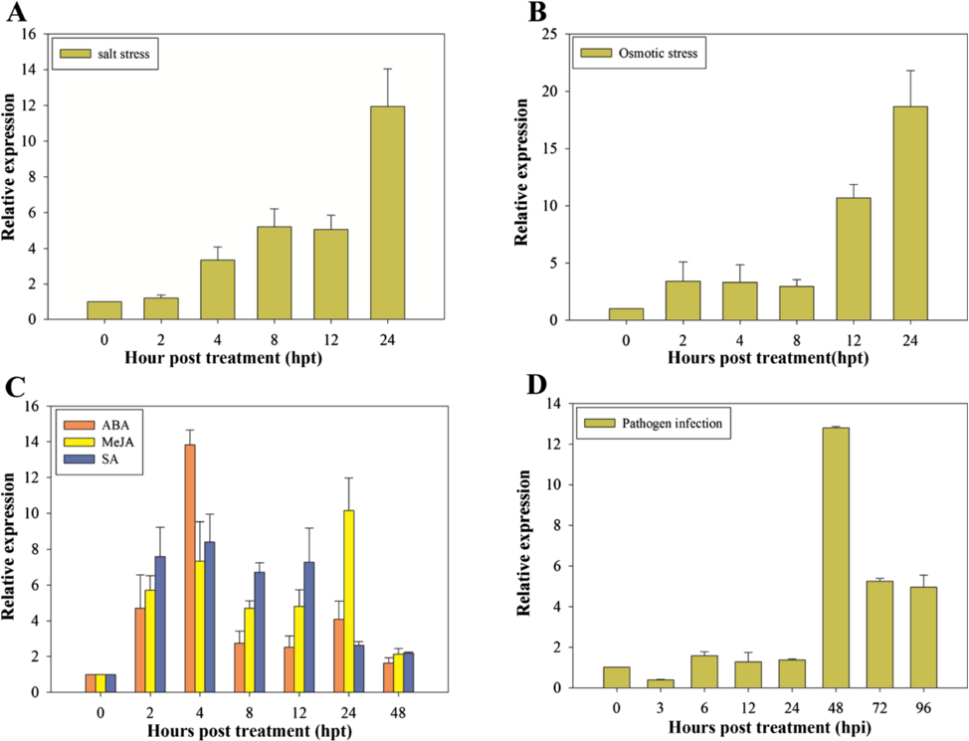
*CaPAO* expression patterns in pepper leaves treated with various stresses. **a** Salt stress; **b** osmotic stress; **c** phytohormone treatments (including ABA, MeJA and SA); **d** *P. capsici* infection. The relative transcriptional expression of *CaPAO* was calculated in various treated leaves in comparison to that in the mock controls across time points. Error bars represent SD for three independent replicates

To analyze the response of the *CaPAO* gene to abiotic stresses, pepper plants were exposed to 400 mM sodium chloride (NaCl) and 400 mM mannitol, and then the abundance of *CaPAO* transcripts was analyzed by quantitative RT-PCR. As shown in Fig. 4a, the *CaPAO* expression level in plants treated with 400 mM NaCl began to increase gradually at 4 h and peaked (11.9-fold) at 24 h, compared to the control (0 h). With osmotic treatment, *CaPAO* was induced quickly in pepper plants (Fig. 4b) with a 3.4-fold increase in expression at 2 h and a stronger 10.7-fold elevation at 12 h. The highest transcript level was detected at 24 h (18.7-fold). These results indicate that the increased abundance of the *CaPAO* gene transcript may be part of the response to abiotic stresses, including high salinity and osmotic stress.

To examine whether stress-related signaling molecules can induce *CaPAO* expression, three phytohormones were used to treat pepper leaves. As shown in Fig. 4c, the expression levels of *CaPAO* in leaves sprayed with abscisic acid (ABA), methyl jasmonate (MeJA) and salicylic acid (SA) were elevated to different extents. ABA could induce quickly the expression of the *CaPAO* gene within the first 4 h after treatment. Compared to the control, *CaPAO* transcripts were detected at the highest level (13.8-fold) at 4 h and then decreased gradually after 8 h. Interestingly, there was a slight up-regulation at 24 h. Spraying pepper plants with MeJA caused a gradual upregulation of *CaPAO* within the first 4 h, but then it declined slowly. However, the *CaPAO* expression peaked by 24 h at a 10.1-fold greater level than the control. By contrast, the treatment of SA quickly induced the *CaPAO* transcript abundance at 2 h to 7.6-fold higher than the control and maintained a relatively steady level from 4 h to 12 h before a sharp downregulation at 24 h. At 48 h, the *CaPAO* transcript decreased to nearly the same levels among all of the plants treated with the three phytohormones. These results indicated that the *CaPAO* gene could be induced and upregulated by all three stress-related signaling molecules tested (ABA, MeJA and SA).

*CaPAO* expression was enhanced in pepper plants infected with *P. capsici* as shown in Fig. 4d*.* The *CaPAO* transcriptional level was slightly decreased at 3 h and remained at a steady level from 6 h to 24 h, compared with the mock control. This transcript then increased rapidly and peaked (12.8-fold) at 48 h. Subsequently, the *CaPAO* expression level sharply downregulated at 72 h (5.3-fold) and decreased to 4.9-fold at 96 h, which was the lowest level after infection relative to the control. These results revealed that *CaPAO* may be involved in the pepper defense response against pathogens.

### VIGS assay of *CaPAO* Gene in pepper plants

#### Silencing efficiency of CaPAO gene

**Fig. 5:**
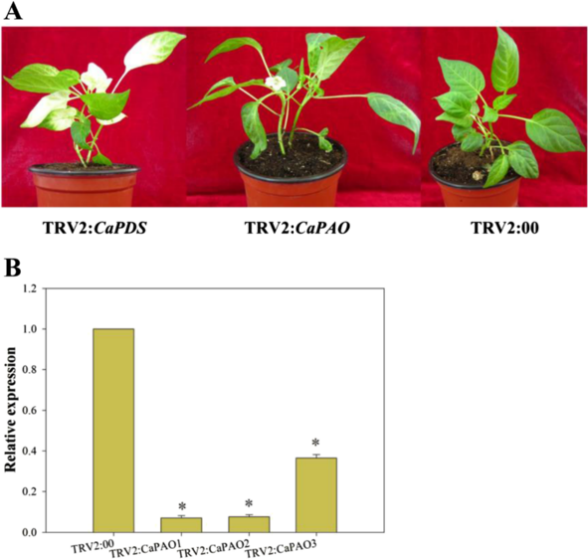
Efficiency of *CaPAO* gene-silencing in pepper plants. **a** Phenotypes of gene-silenced pepper plants 45 d after inoculation. Left: control plant (TRV2:00); middle: *CaPDS*-silenced plant (TRV2: *CaPDS*); right: *CaPAO*-silenced plant (TRV2: *CaPAO*). **b** Quantitative real time-PCR analysis of *CaPAO* expression levels in leaves of *CaPAO*-silenced plants (TRV2: *CaPAO1*, TRV2: *CaPAO2* and TRV2: *CaPAO3*) and control plants (TRV2:00) 45 d after inoculation. Error bars represent SD for three independent replicates. Asterisks indicate a significant difference (*p* < 0.05) compared to TRV2:00 leaves

The results showed that *CaPAO* participates in the chlorophyll degradation pathway and is involved in the response to various stresses in pepper plants. To further examine the function of *CaPAO* in pepper, a tobacco rattle virus (TRV)-based VIGS technique was used. Pepper plants inoculated with *Agrobacterium* for 6–7 weeks were used for the following treatments. An empty vector was applied to plants (TRV2:00) as a negative control. TRV2: *CaPDS* plants, in the endogenous *phytoene desaturase* (*PDS*) gene was silenced to cause photobleaching, were used as positive controls for testing VIGS efficiency. Three weeks after the *Agrobacterium* inoculation, we found that most of the plants clearly showed symptoms of viral infection. Furthermore, the TRV2: *CaPDS* plants began to exhibit the photobleached phenotype. These results indicated that VIGS was successfully applied in this experiment. As shown in Fig. 5a, no morphological distinction was observed between the *CaPAO*-silenced plants (TRV2: *CaPAO*) and the empty vector treated control plants (TRV2:00) 45 d after inoculation. Simultaneously, *CaPAO* transcript levels in empty vector control plants (TRV2:00) and *CaPAO*-silenced plants (TRV2: *CaPAO*) were examined by quantitative RT-PCR to screen the efficiency of *CaPAO* gene silencing by VIGS (Fig. 5b). The results showed that the *CaPAO* transcriptional level was reduced remarkably in *CaPAO*-silenced plants compared to the empty vector control, which demonstrated that the gene silencing was successful.

#### Silencing of CaPAO delay chlorophyll breakdown under high salt treatment

**Fig. 6:**
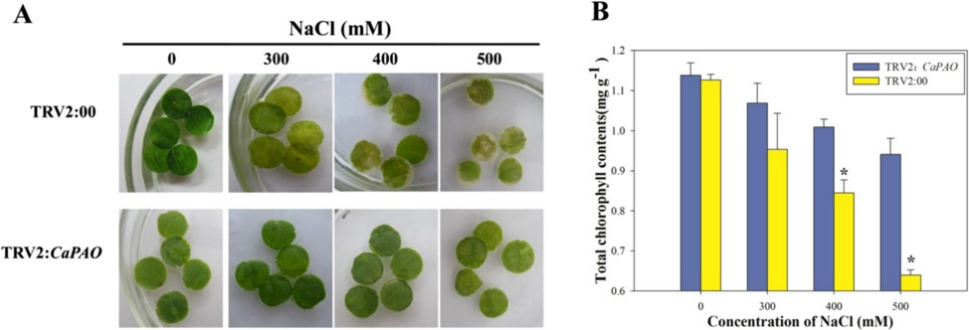
Chlorophyll degradation in leaves of gene-silenced pepper plants. **a** Phenotypes and **b** Total chlorophyll contents of leaf discs of the gene-silenced plants in response to high salt stress. Leaf discs from the gene-silenced pepper leaves were floated in different concentrations of NaCl solutions (0, 300, 400, and 500 mM)with continuous fluorescent lighting at 25 °C for 3 days. Error bars represent SD for three biological replicates. Asterisks indicate a significant difference (*p* < 0.05) compared to TRV2:00 leaves

In order to analyse whether the *CaPAO* response to salt stress, the leaf discs from empty vector control-treated (TRV2:00) and *CaPAO*-silenced (TRV2: *CaPAO*) plants were exposed to various concentrations (0, 300, 400 and 500 mM) of NaCl solution with continuous lighting for 3 d. As shown in Fig. 6a, the color of the leaf discs of TRV2:00 plants turned yellow under 300 mM NaCl treament and the leaf discs appeared to be a bleached phenotype under treatment with higher NaCl concentrations (400 and 500 mM). However, the leaf discs of TRV2: *CaPAO* plants slightly shrinked and their color showed little change. Furthermore, chlorophyll breakdown in TRV2:00 leaves was observed to occur faster than that in TRV2: *CaPAO* leaves 3 d after treatment with high NaCl concentrations (400 and 500 mM) (Fig. 6b). These results suggested that silencing the *CaPAO* gene can inhibit chlorophyll breakdown during salt stress-induced leaf senescence in pepper plants.

### Effect of *RD29A* promoter-inducible overexpression of *CaPAO* on salt tolerance of tobacco

#### Identification of transgenic plants

To examine the relative contribution of *CaPAO* to salt stress-induced leaf senescence, tobacco plants were transformed with PVBG2307-PRD29A-*CaPAO* vector. A sequence of 491 bp, which contained a 284-bp fragment of the RD29A promoter and a 207-bp fragment of the *CaPAO* gene, was produced from genomic DNAs of the positive transgenic T_0_ lines. Nine plants were confirmed to be positive transgenic T_0_ lines. T_1_ and T_2_ generation of transgenic tobacco plants were selected using 50 mg · L^−1^ kanamycin, and finally obtained transgenic homozygous T_2_ lines. One T_2_ positive transgenic plants were used in subsequent studies.

#### Semi-quantitative RT-PCR analysis of the transgenic tobacco plants after salt stress treatment

**Fig. 7:**
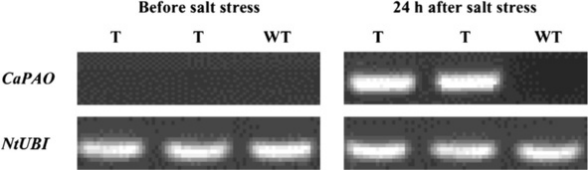
RT-PCR analysis of *CaPAO* in transgenic tobacco plants with and without salt treatment under 150 mM NaCl. T: T_2_ generation of transgenic tobacco plants, WT: wild type (non-transgenic tobacco plants)

As shown in Fig. 7, *CaPAO* showed no expression in the *CaPAO* transgenic tobacco and wild type (WT) plants before salt stress treatment. *CaPAO* was strongly induced in the leaves of the transgenic plants at 24 h after treatment with 150 mM NaCl solution, while it was not expressed in WT plants at all. This may suggest that the *CaPAO* gene, controlled under the stress-inducible promoter RD29A, was induced to express under exogenous salt treatment in positive transgenic tobacco plants.

#### Phenotype of transgenic tobacco plants after salt stress treatment

**Fig. 8:**
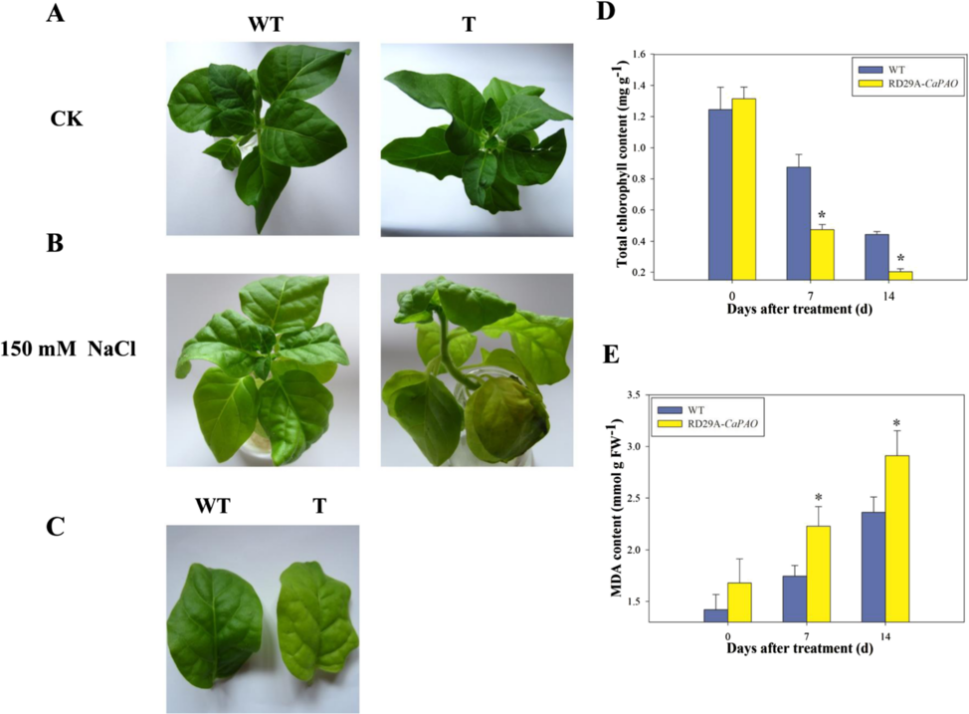
Phenotypes (**a**, **b**, **c**), chlorophyll contents (**d**) and MDA contents (**e**) of transgenic tobacco plants after salt treatment under 150 mM NaCl. **a** Phenotypes of wild type and transgenic plants before salt stress, **b** phenotypes of wild type and transgenic plants after salt treatment for 7 d, **c** phenotypes of leaves of wild type and transgenic plants after salt treatment for 7 d, **d** chlorophyll contents and **e** MDA contents of transgenic and wild-type tobacco plants under salt stress for 7 and 14 d, respectively. Error bars represent SD for three biological replicates. Asterisks indicate a significant difference (*p* < 0.05) compared to wild type leaves

Before salt stress treatment, there were no phenotypic differences between *CaPAO* transgenic and WT plants (Fig. 8a). At early stage of the salt stress treatment (150 mM NaCl solution), both leaves of transgenic and WT plants wilted with more serious wilting degree in the WT plants. Along with the time of treatment, the lower part of leaves became yellow. After salt treatment for 7 d, the leaves of the transgenic plants became yellow and the edge of the lower leaves showed necrotic symptoms after salt stress treatment, while the upper young leaves were green. However, leaves of WT plants appeared to shrink, with lower leaves turning yellow but upper leaves remaining green (Fig. 8b-c).

#### Chlorophyll and Malondialdehyde (MDA) contents of transgenic plants after treatment with salt stress

Leaf senescence is associated with chlorophyll breakdown and reactive oxygen species (ROS) accumulation [19, 20]. ROS-generated lipid peroxidation (reflected by MDA content) is an inherent feature of senescing cells and a source of ROS [21, 22]. To further analyze the effect of *CaPAO* overexpression on salt-induced leaf senescence in tobacco, chlorophyll and MDA contents were measured after salt treatment for 7 and 14 days, respectively (Fig. 8d-e). Before the salt stress, there were few obvious differences in chlorophyll and MDA content between WT and transgenic leaves. Chlorophyll content of WT and transgenic leaves decreased by 0.37 and 0.84 mg · g^−1^ after 7 days, and decreased by 0.44 and 0.20 mg · g^−1^ after 14 days of salt treatment, respectively. The rate of chlorophyll breakdown in WT plants was higher than that in the transgenic plants. Correspondingly, MDA content gradually increased with elongated treated time. MDA content of WT and transgenic leaves increased by 0.32 and 0.55 mmol · g ^-1^ after 7 days, and increased by 2.36 and 2.91 mmol · g ^-1^ after 14 days of salt treatment, respectively. MDA content in WT plants was significantly lower than that in transgenic plants. These results indicated that overexpression of *CaPAO* could accelerate the chlorophyll breakdown and lipid peroxidation accumulation of transgenic tobacco plants under salt treatment. All these observations suggested that overexpression of *CaPAO* could accelerate salt-induced leaf senescence in tobacco.

## Discussion

PAO opens the porphyrin macrocycle of pheophorbide *a* and finally forms FCCs [3]. Here, we identified the *CaPAO* gene from pepper. CaPAO contained conserved domains that existed in PAO homologues. Furthermore, CaPAO shared high homology to other PAO proteins, especially SlLLS1 and NtPAO. Bioinformatics analyses demonstrated that CaPAO should function as a PAO.

Tissue-specific analysis showed that *CaPAO* was expressed in all tissues, and the expression level in leaves was higher than other tissues. The findings were similar to the previous results in maize and rice [8, 23], it was reported that expression of the *OsPAO* gene was detected in all tissues and at the highest level in leaves. However, the present results were different than those in *A. thaliana* [12], in which *AtPAO* transcript was higher in flowers and siliques than other tissues. These results revealed that *PAO* is important to growth and development of all tissues, although its function may be distinct in different species.

Various biotic and abiotic stresses can affect growth and development of pepper plants, causing chlorophyll breakdown, cell death and finally premature senescence [1, 6, 24, 25]. All of these consequences would impact the yield and quality of crop plants. PAO is an important intermediate in chlorophyll degradation, the expression and activity of which can be affected by various stresses. Wang *et al.* (2012) reported that relative expression of and *PAO* in drought-stressed leaves was greatly upregulated in apple [26]. Microarray analysis indicated that upregulation of PAO in response to various stress conditions, coinciding with breakdown of Chlorophyll under these conditions [3, 27]. Results of the current study showed that transcript levels of the *CaPAO* gene were upregulated in pepper plants after treatment with various stresses, including high salinity, osmosis and *Phytophthora capsici* infection. These results suggested that *CaPAO* may be involved defense response to salt and osmotic stresses as well as *Phytophthora capsici* in pepper plants.

Previous studies have shown that phytohormone signaling pathways played an important role in mediating developmental processes and environmental responses in plants [2, 28]. These hormones could cause the expression of stress-related genes, which in turn affect senescence progress of leaf. Many studies have proved that *CaPAO* gene expression or activity was regulated by hormones [29–32]. For instance, ABA can enhance the expression of *PAO* in rice leaves [29]. Rodoni *et al.* (1998) suggested that the chlorophyll breakdown related to PAO activity in barley detached leaves, a rapid loss of chlorophyll in conjunction with an increase of PAO activity was occurred after treatment with ABA and MeJA [30]. In the current study, treatment with ABA, MeJA or SA was found to increase the expression level of *CaPAO*. These findings suggested that the *CaPAO* gene may be involved in ABA-, JA- and SA-dependent signaling pathways. These results were different from those of a study in wheat reporting that ABA and MeJA enhanced the expression of *TaPAO*, while SA treatment did not cause significant changes in the level of this gene [6]. SA is the hormone involved in pathogen response and abiotic stress tolerance in pepper plant [22, 33]. In perennials, SA may be involved in the regulation of drought-induced leaf senescence as SA accumulation preceded chlorophyll breakdown and nitrogen mobilization [34]. Here, high salinity, osmosis and *Phytophthora capsici* infection induced *CaPAO* expression in pepper leaves. It is likely that salt, mannitol and *Phytophthora capsici*-induced SA accumulation may trigger *CaPAO* transcripts in pepper plants.

The function of the *PAO* gene has been studied in many plants [8, 10, 35]. Tang *et al.* (2011) reported that chlorophyll breakdown was delayed compared to controls in leaves detached from leaves of PAO-silenced rice plants during dark-induced senescence [8]. Moreover, leaves with overexpression of *PAO* in *Arabidopsis* had lower chlorophyll content and earlier leaf yellowing than the control 4 d after dark treatment [35]. Leaf senescence was involved in chlorophyll degradation and accumulation of ROS. Drought-induced senescence in the leaves of apple was reflected in chlorophyll loss and an increase in the levels of ROS, with the induction of the *PAO* gene [26]. In this research, the delay of chlorophyll degradation was detectable in the detached leaves from *CaPAO* silencing pepper plants 3 days after treatment with salt. Conversely, overexpression of *CaPAO,* induced by the stress-inducible promoter RD29A, could accelerate the rate of chlorophyll breakdown and lipid peroxidation of the transgenic plants under salt stress. The results of Tang *et al*. (2013) have shown that overexpressing *TaPAO* caused an accumulation of RCCs in wheat leaves [36]. RCCs have been proved to be phototoxic and caused cell deach, resulting in senescence [8, 37]. All of these results demonstrated that *CaCP* played crucial role in pepper plant defense response to salt stresses, thus delaying leaf senescence.

## Conclusions

Overall, we conclude that *CaPAO* plays an important role in senescence and chlorophyll degradation, as well as defense responses to various stresses. *CaPAO* was induced in natural senescence and various stresses. Silence of *CaPAO* resulted in a stay-green phenotype in pepper plants, and overexpression of *CaPAO* accelerated the process of salt-induced leaf senescence. In the future, the transgenic plants about overexpression or knockdown of *CaPAO* gene in pepper would be used for studying detail function of *CaPAO*.

## Methods

### Plant material

The B12 pepper cultivar was used for cloning and characterization of the *CaPAO* gene in this study. This early maturing variety was selected by a pepper research group in the College of Horticulture, Northwest A&F University, China. The seeds were treated with warm water (55 °C) for 20 min and then incubated at 28 °C to accelerate germination under dark conditions. The seeds were rinsed twice every day until budding. The germinated seeds were sown in pots containing compost. Seedlings were placed in a growth chamber for a 16-h light/8-h dark cycle at 25 °C/21 °C, respectively.

### Cloning and sequence analysis of *CaPAO* gene

To clone the *CaPAO* gene, total RNA was extracted from green mature leaves using the Trizol (Invitrogen) method, and first-strand cDNA synthesis was performed using Smart RACE cDNA amplification kit (Clontech). The reported nucleotide sequence of *Nicotiana tabacum PAO* (accession number: EU294211.1) was used as the query probe to retrieve homologous expressed sequence tag (EST) sequences of pepper in GenBank using the BLASTN protocol. Four overlapping pepper ESTs (accession numbers: GD077816, GD078012, GD072064 and GD077857) were chosen to be assembled into a splicing fragment without a complete ORF. To confirm the authenticity of the assembled pepper sequence, the EST-PAOF/EST-PAOR primer pair was used (Table 1). The design of both primers was based on the splicing sequence. The PCR reaction conditions were as follows: 94 °C for 5 min; 35 cycles at 94 °C for 30 s, 61 °C for 30 s, 72 °C for 1.5 min; and finally an extension at 72 °C for 10 min. The product was obtained, subcloned into a pMD19-T vector (TaKaRa) and sequenced.

**Table 1:** Primer sequences in this study

primer	Sequence (5′-3′)
Cloning of CaPAO gene	
EST-*CaPAO*F	CTTCACTCCCAATCACCAAACC
EST-*CaPAO*R	TCGCTAACGAGCCCAAGATAAT
5′RACE-*CaPAO*GSP	CGGAGACTGAAACGCTTTAGCTTC
5′RACE-*CaPAO*NGSP	GAACAACCCACTGAGACCCAGATTT
3′RACE-*CaPAO*GSP	TTGGTGGCAGGTTTTTCCGAGAT
3′RACE-*CaPAO*NGSP	CGGCAGCACAGACAACCAACCAC
quantitative real-time PCR	
RT-*CaPAO*F	CAAGTTTGCCTACACCATTTCAG
RT-*CaPAO*R	GCCCATTTTCATCTAATCTCCCT
*CaUBI3*F	TGTCCATCTGCTCTCTGTTG
*CaUBI3*R	CACCCCAAGCACAATAAGAC
Virus-induced gene silencing (VIGS) vector construction	
TRV-*CaPAO*F	CAATGGCACCAGGAAAGACC
TRV-*CaPAO*R	GCGTTTGGACAAGACGGTAG
*CaPDS*F	TGTTGTCAAAACTCCAAGGTCTGTA
*CaPDS*R	TTTCTCCCACTTGGTTCACTCTTGT
Tabacco genetic transformation	
2307-*CaPAO*F	ATGGCTTCTTCTTCTTCTTGTAT
2307-*CaPAOR*	TTCTAAATTTCCCTAGACTTCTAGTC
RD29A-F	GAATAAATATCATACCGACATCA
*CaPAO*-R	CAGTACTAGTAGATGATTCTTGTTC
*NtUBIF*	TCCAGAAAGAGTCAACCCG
*NtUBIR*	GACCTCAGTAGACAAAGCACAT

To isolate a full-length cDNA of the putative *CaPAO* gene with complete 5’ and 3’ regions, the RACE method was used. Gene-specific primers (5′-GSP, 5′-NGSP, 3′-GSP, and 3′-NGSP) were designed according to the partial cDNA sequence (Table 1). The 5′-GSP (external) and 5′-NGSP (internal) primers were used to isolate 5’-end sequences, while the 3’-GSP (external) and 3’-NGSP (internal) primers were used for isolation of 3’-end sequences. Nested PCR was performed in the 5′RACE and 3′RACE procedures. The amplified PCR products were ligated into the pMD19-T vector and sequenced. Finally, all acquired sequences were assembled into a single sequence with a complete ORF using the Contig Expression software and BLAST online software (http://www.ncbi.nlm.gov/blast).

The putative *Ca*PAO cDNA and protein sequences were analyzed by the DNAMAN software 5.2.2 and BLAST online software. Prediction of the subcellular localization of the putative signal peptide, chloroplast transit peptides and its cleavage site were carried out using the CBS prediction server’s online program (http://www.cbs.dtu.dk/services/). The p*I* and MW of the putative protein were analyzed with the p*I*/MW program (http://www.expasy.org/), and its secondary structure was predicted using the Scratch Protein Predictor online program (http://scratch.proteomics.ics.uci.edu). The multiple sequence alignments of *Ca*PAO and other PAO proteins were performed with DNAMAN software. The phylogenetic tree was constructed using the MEGA5.05 program with the neighbor-joining method.

### *CaPAO* Gene expression pattern analysis

#### Tissue-specific expression of CaPAO gene

To evaluate the *CaPAO* gene expression levels in different tissues, root, stem, leaf and flower samples were collected from the untreated B12 pepper cultivar. To examine *CaPAO* expression pattern during leaf development, leaves were harvested at young, fully expanded and senescent stage, respectively. All samples were frozen in liquid nitrogen and kept at −80 °C prior to RNA extraction.

#### Stress treatments

Signaling molecules as well as abiotic and biotic stresses were used to treat six-leaf stage pepper plants. For treatments with signaling molecules, plant hormones were applied by spraying pepper plant leaves with either 0.57 mM ABA, 5 mM SA or 1 M MeJA dissolved in 0.05 % (v/v) ethanol. The control plants were treated with distilled water containing 0.05 % (v/v) ethanol. After 0, 2, 4, 8, 12, 24 and 48 h of treatment, pepper leaves were sampled. For salt and drought stress treatments, the pepper plants were uprooted from the soil, and their roots were soaked in 400 mM NaCl and 400 mM mannitol, respectively. The control seedlings were treated with sterile water. After 0, 2, 4, 8, 12 and 24 h of treatment, pepper leaves were collected. For the fungal pathogen treatment, pepper plants were inoculated with *Phytophthora capsici* (HX-9 strain) by the root-drenching method, while control plants were treated with sterile distilled water [38]. The inoculated and control plants were incubated in a growth chamber at 28 °C under a 16-h light/8-h dark photoperiod cycle with 60 % relative humidity. The samples were collected at 0, 3, 6, 12, 24, 48, 72 and 96 h intervals. All samples were frozen immediately in liquid nitrogen and kept at −80 °C prior to RNA extraction.

### Isolation RNA and genomic DNA

Total RNA was extracted from pepper and tobacco using the Trizol (Invitrogen) method. Contaminated genomic DNA was digested by RNase-free DNase I (Promega). The concentration and purity of total RNA were determined spectrophotometrically using a NanoDrop instrument (Thermo Scientific NanoDrop 2000C Technologies). The first-strand cDNA was synthesized according to the instructions of the PrimeScript™ Kit (TaKaRa). Genomic DNA was extracted from tobacco mature leaves using CTAB method.

### Quantitative real-time PCR and semi-quantitative RT-PCR analysis

In order to identify the *CaPAO* expression patterns in different pepper tissues without treatment and in leaves under various stresses, qRT-PCR was performed using SYBR® Premix Ex Taq™ II (TaKaRa) in 20 μl reaction volume containing 10.0 μl SYBR® Premix Ex Taq™ II, 2.0 μl diluted cDNA, and 0.8 μl of forward and reverse primers. The amplification was completed with the following cycling parameters: 95 °C for 1 min, followed by 40 cycles of 95 °C for 10 s, 57 °C for 20 s and 72 °C for 20 s. The *CaUBI3* gene (accession number: AY486137.1) was used as an internal control (reference gene) in this study (Table 1). The relative gene expression levels were calculated using the 2^−ΔΔCt^ comparative threshold method [39]. All samples were performed in triplicate, and each had at least three independent biological replicates.

Semi-quantitative RT-PCR was performed for analysis the expression level of *CaPAO* gene in transgenic tobacco, and the *RD29A*-F and *CaPAO*-R primer pair were used. The PCR cycles were 1 min at 95 °C followed by 29 cycles at 30 s at 95 °C, 30 s at 53 °C, and 60 s at 72 °C followed by an extension for 10 min at 72 °C. The PCR products were separated on 2 % agarose gels stained with ethidium bromide to detect expression degree of *CaPAO* gene. The *NtUBI* gene was used as a reference gene.

### Plasmid construction and inoculation of VIGS agrobacterium in pepper plants

#### Construction of VIGS Plasmids TRV2-CaPAO and TRV2-CaPDS

A *CaPAO* sequence fragment was amplified using gene-specific primers with restriction sites *Xba*I (forward) and *Bam*HI (reverse) and inserted into the pTRV2 vector to generate TRV2-*CaPAO*. The primers for VIGS are shown in Table 1. The *CaPDS* gene (*phytoene desaturase* from *C. annuum*, accession number: X68058.1) was used for determining the effectiveness of VIGS in this study, and the TRV2-*CaPDS* vector was designed by our laboratory. Finally, these plasmids (pTRV1, pTRV2, TRV2-*CaPDS* and TRV2-*CaPAO*) were each transformed into the *Agrobacterium tumefaciens* strain GV3101.

#### Inoculation of VIGS Agrobacterium in pepper plants

Preparation of *A. tumefaciens* harboring pTRV1, pTRV2, TRV2-*CaPDS* or TRV2-*CaPAO* vectors were described by Liu et al. [40]. Before inoculation, cultures containing pTRV1 and pTRV2 or their derivatives (TRV2-*CaPDS* or TRV2-*CaPAO*) were mixed at a 1:1 ratio. The mixtures of *Agrobacterium* were inoculated into the fully-expanded cotyledons of the B12 pepper cultivars. Plants were placed in a growth chamber at 18 °C and 60 % relative humidity for 2 d. Subsequently, the plants were grown at 23 °C under a 16-h light/8-h dark photoperiod cycle and 60 % relative humidity. At 45 days after inoculation, the leaves were used for characterization and analysis of *CaPAO.*

#### Treatment of CaPAO-silenced plants

To analyse salt stress of the gene-silenced plants, the leaf discs from empty vector control-treated (TRV2:00) and *CaPAO*-silenced (TRV2: *CaPAO*) plants were floated in various concentrations (0, 300, 400 and 500 mM) of NaCl solution with continuous lighting at 25 °C for 3 days. The symptoms of leaf discs were observed during treatment, and the chlorophyll content was determined after 3 d.

### Tobacco genetic transformation of the *CaPAO* gene

#### Construction of transgenic vector PVBG2307-RD29A-CaPAO

The *CaCP* sequence with a complete ORF was amplified using a primer pair 2307-*CaPAO*F/2307-*CaPAO*R and inserted into PVBG2307 plasmid possessing *Bam*HI and *Kpn*I restriction enzyme sites to yield PVBG2307-*CaPAO.* Then the *RD29A* promoter sequence from RD29A-T vector was cloned into PVBG2307-*CaPAO* vector using digestion of *Hin*dIII and *Xba*I restriction enzymes. PVBG2307-PRD29A-*CaPAO* vector was constructed successfully. Finally, the plasmid was transformed into the *Agrobacterium tumefaciens* strain GV3101.

#### Plant transformation and selection of T_2_ generation of transgenic tobacco plants

Tobacco plants (*Nicotiana tabacum* cv. Bairihong) were used for all subsequent analyses, the successful transformed plants with leaf disc method were selected and incubated as previously described by Li et al. [41]. The primers RD29A-F and *CaPAO*-R were used to conform the transgenic tobacco seedlings at genomic DNA level, and a second pair of primers, 2307-*CaPAO*F and *CaPAO*-R were used to further confirm the transgenic status of transgenic tobacco plants. Growth conditions of tobacco seedlings and seed selection were followed according to the method of previous research [42]. The T_2_ offspring of transgenic tobacco plants were used for all subsequent experiments.

#### Treatment of transgenic tobacco plants

Seedlings of WT and one tobacco line (T) at the stage of four true leaves were carefully removed from the pots and their roots washed with tap water prior to solution culture in 1/4 strength Hoagland’s solution. After seven to eight true leaves stage, the seedlings were cultured with 1/2 Hoagland’s solution supplied with 150 mM NaCl solution. Leaves were harvested at 0, 7, and 14 d after salt treatment, and total chlorophyll and MDA contents were measured. All treatments were repeated three times and arranged in a randomized complete block design.

### Determination of chlorophyll content and MDA content

For determination of chlorophyll content, leaf samples (0.5 g) were grinded into a fine powder in the presence of liquid nitrogen, mixed with 8 ml acetone 80 % (v/v) and kept overnight at 4 °C to extract chlorophyll. The supernatant containing chlorophyll was obtained after centrifugation at 10,000 × *g* for 10 min at 4 °C. The chlorophyll content was measured by a spectrophotometric method, and results were expressed in milligrams of total chlorophyll per gram fresh weight of tissues [43]. The MDA content of treated leaves was measured according to the method of Buege and Aust [44]. All experiments were replicated three times.

### Primers used in this study

Primers used for cloning, quantitative real-time PCR analysis, semi-quantitative RT-PCR Analysis, VIGS and transgenosis are listed in Table 1.

### Statistical analysis

All data are presented as the mean ± standard deviation (SD) of three replicates. Quantitative data were analyzed using Statistical Analysis System software (SAS Institute, version 8.2) following one-way analysis of variance (ANOVA). Differences among means were analyzed at a significance level of 0.05.

## Abbreviations

ABA: Abscisic acid
bp: base pair(s)
DREs: Dehydration- responsive elements
EST: Expressed sequence tag
FCC: Fluorescent chlorophyll catabolite
MDA: Malondialdehyde
MeJA: Methyl jasmonate
MW: Molecular weight
NaCl: sodium chloride
ORF: Open reading frame
PAO: Pheophorbide *a* oxygenase
pheide *a*: pheophorbide *a*
p*I*: isoelectric point
qRT-PCR: quantitative real-time PCR
RACE: Rapid amplification of cDNA ends
SA: Salicylic acid
TRV: Tobacco rattle virus
UTR: Untranslated region
VIGS: Virus-induced gene silencing
WT: Wild- type
